# Microbes-mineral interactions enhance nutrient acquisition and physiological adaptation in *Glycine max* under Sulfur-deficient conditions

**DOI:** 10.3389/fpls.2026.1723856

**Published:** 2026-07-10

**Authors:** Pratibha Verma, Priyanka Chauhan, Navinit Kumar, Nishtha Mishra, Sahil Mahfooz, Vartika Gupta, Aradhana Mishra, Lal Bahadur

**Affiliations:** 1 Division of Microbial Technology, Council of Scientific and Industrial Research (CSIR)-National Botanical Research Institute, Lucknow, Uttar Pradesh, India; 2 Academy of Scientific and Innovative Research (AcSIR), Ghaziabad, India; 3 Soil Science Laboratory, Council of Scientific and Industrial Research (CSIR)–National Botanical Research Institute, Lucknow, India; 4 School of Sciences, P P Savani University, Kosamba, Surat, India; 5 Department of Chemistry, Deen Dayal Upadhyaya Gorakhpur University, Gorakhpur, India; 6 Department of Industrial Microbiology, Deen Dayal Upadhyaya Gorakhpur University, Gorakhpur, India; 7 Division of Environmental Technologies, Council of Scientific and Industrial Research (CSIR)–National Botanical Research Institute, Lucknow, India

**Keywords:** gene expression, microbial intervention, nutrient acquisition, sulfur deficiency, *Trichoderma reesei*

## Abstract

**Introduction:**

Sulfur is finite source for plants to develop their growth and productivity as well as help in plant's metabolism. Its deficiency causes the chlorosis, and thin stem in plant. Here in *Trichoderma reesie* (TP-5) was used to compared with other microbes for solubilization of nutrients during sulfur deficiency in soybean.

**Methods:**

Sulfur solubilizing activity and plant growth-promoting traits were performed *in vitro*. Further plants were inoculated with microbes under sulfur deficiency to identify the nutrient acquisition, SEM analysis and gene expression.

**Results:**

The *in vitro* study results showed that TP-5 has higher sulfur solubilization as well as plant growth-promoting ability. Moreover, in *in vivo* experiment TP-5 enhances photosynthetic pigment soybean under 25% sulfur deficient condition. Additionally, different stress ameliorating enzymes *viz*. SOD, CAT, APx, and PPO were also enhanced in TP-5 treated soybean root and shoot. Similarly, macronutrients such as N, P, S, Ca, and K and micro-nutrients B, Co, Cr, Zn, Ni, Fe, and Cu increase in TP-5+25%S treated soybean. The results were further confirmed by qRT-PCR assay, which showed that sulfur metabolism related genes *i.e., SULTR1, SAT 2, ATPS, NIR, DES*, and *GR* were highly upregulated in TP-5+25%S treated soybean. Moreover, the colonization of TP-5 in soybean tissue was also validate by SEM analysis.

**Discussion:**

Conclusively, present investigation gives evidence that TP-5 is efficient to solubilize sulfur, balance the nitrogen and sulfur ratio during abiotic stresses especially nutrient deficient condition.

## 1 Introduction

Sulfur (S) deficiency has increasingly been identified as a ‘hidden but essential constraint’ in intensive agricultural systems around the world. The shift towards the application of high-analysis, Sulfur-free fertilizers, in addition to lower application rates of organic manure and continuous cropping, has led to a considerable decrease in soil Sulfur pools (Dobrokhotov et al., 2023). As a macronutrient, Sulfur is a vital constituent of primary and secondary metabolism and acts as a structural part of cysteine, methionine, glutathione, sulfolipids, and different coenzymes involved in redox regulation and cell homeostasis (Kopriva et al., 2019). Sulfur deficiency affects assimilatory sulphate reduction reactions, chlorophyll biosynthesis, nitrogen-Sulfur ratios, and oxidative stress tolerance. Notwithstanding the agronomic significance of Sulfur, its management is mostly dependent on chemical fertilization, which frequently leads to nutrient leaching, soil acidification, and eutrophication of ecosystems (Pahalvi et al., 2021). Hence, there is an urgent need for the development of sustainable and biologically integrated approaches to enhance Sulfur availability and utilization efficiency.

Among the economically important crops, soybean (*Glycine max* L.) is more responsive to Sulfur deficiency due to its high protein synthesis (35-40%) and high demand for sulfur-containing amino acids (Balboa et al., 2018). Soybean productivity and seed nutritional quality are directly linked to efficient Sulfur assimilation and its coordination with nitrogen metabolism. Sulfur deficiency in soybean results in chlorosis of young leaves, decreased nodulation and biological nitrogen fixation, delayed reproductive growth, and decreased seed protein concentration (Dawar et al., 2023; Sohail et al., 2024). At the molecular level, Sulfur status regulates the expression of sulfate transporter genes belonging to the SULTR gene family and major enzymes such as ATP sulfurylase, APS reductase, and sulfite reductase, which together regulate the metabolic flux through the assimilatory pathway. Moreover, Sulfur-derived thiol compounds such as glutathione play a crucial role in reactive oxygen species (ROS) detoxification and stress tolerance (Sachdev et al., 2021). Regardless of the elaborate adaptation by the soybean to deal with sulfur deficiency, it is important to improve the efficacy in the process of sulfur uptake and redox balance under sulfur-deficient soil conditions (Dawar et al., 2023).

In this context, rhizosphere-associated microorganisms represent a promising ecological strategy for Sulfur bioavailability restoration. Microbial oxidation of reduced Sulfur compounds (such as elemental Sulfur and thiosulphate) to plant-available sulphate forms is a major biogeochemical process catalyzed by Sulfur-oxidizing bacteria and fungi (Ranadev et al., 2023; Zhao et al., 2017; Sun et al., 2024). Besides nutrient transformation, plant growth-promoting fungi such as *Trichoderma* spp. have been demonstrated to enhance root system architecture, boost nutrient transporter gene expression, and trigger systemic tolerance mechanisms (Harman, 2024). Recent studies have shown that certain strains of fungi are responsible for increasing sulfur mobilization, along with changes in plant physiology and molecular mechanisms in response to nutrient stress; yet, the mechanism by which these changes occur and knowledge about sulfur mobilization by fungi are still unclear (Xu et al., 2018; Mitra, 2018). However, despite these recent observations, the comprehensive understanding of Sulfur-mobilizing fungi in soybean under Sulfur deficiency.

Considering our previous results on the adaptive responses of *Trichoderma reesei* to nutrient-stress situations (Singh et al., 2019), along with their known effectiveness for mobilization of sulfur from soil sources, we proposed that they might be useful for promoting sulfur nutrition and stress tolerance in soybean plants experiencing sulfur-deficiency stress. In view of this hypothesis, the current study involved a comparative evaluation of the effectiveness of selected bacterial isolates and *T. reesei* (TP-5) strains in promoting sulfur acquisition and metabolism, as well as antioxidants production, under different levels of sulfur availability. In order to find out the most appropriate microbial strain for improving sulfur utilization, morphological, physiological, biochemical, and molecular analysis methods were adopted in the study.

## 2 Materials and methods

### 2.1 Chemicals and microbial strain

All the chemicals used in this experiment were of analytical grade and were obtained from HiMedia Laboratories (India) and Sigma-Aldrich (India). The microbial strains were obtained from the laboratory stock of the Division of Microbial Technology, CSIR-National Botanical Research Institute (NBRI), Lucknow, India. Strains B1 to B7 were bacterial endophytes, and TP-5 (MTCC 5659) was the fungal strain used in this experiment. The taxonomic details are given in **Table 1**.

**Table 1:** Microbial strains with accession number.

S. no.	Microbial strains	Source	Accession number
1	*Bacillus amyloliquefaciens* (B1)	*Glycine max*	MT613648.1
2	*Bacillus* spp. (B2)	*Glycine max*	MT623133.1
3	*Brevibacillus brevis* (B3)	*Glycine max*	ON912007.1
4	*Bacillus subtilis* (B4)	*Glycine max*	MH282913.1
5	*Bacillus* sp. (B5)	*Glycine max*	ON912056.1
6	*Bacillus subtilis* (B6)	*Solanum lycopersicum*	MG607368.1
7	*Bacillus amyloliquefaciens* (B7)	*Withania somnifera*	KT962915
8	*Trichoderma reesei* (TP-5)	Protoplast fusion	MTCC5659

### 2.2 Biochemical characterization of microbial strains

#### 2.2.1 Sulfur solubilization

Sulfur-oxidizing activity was determined on sodium thiosulfate broth containing per liter: 5.0 g Na_2_S_2_O_3_, 0.1 g K_2_HPO_4_, 0.2 g NaHCO_3_, 0.1 g NH_4_Cl, and 20 mg bromophenol blue, pH 8.0. 48-hour-old bacterial cultures and 5-day-old fungal cultures were inoculated into broth and incubated at 28 ± 2 °C for 10 days. A change in color from purple to yellow indicated the oxidation of S by the S-oxidizing microbes (Senthilkumar et al., 2021).

#### 2.2.2 Quantification of sulfur

After 10 days of incubation at 30 °C, 1 mL of culture was centrifuged at 15,000 × g for 15 min. The supernatant was diluted (1:10, v/v) with Milli-Q water. One milliliter of 10% BaCl_2_ was added, and turbidity (BaSO_4_ formation) was measured at 450 nm. A standard curve was prepared using potassium sulphate (Kolmert et al., 2000).

#### 2.2.3 Estimation of plant growth-promoting activity

P-solubilization was assessed using Nautiyal’s method (Nautiyal, 1999). Fresh microbial cultures were stabbed onto NBRIP agar plates and incubated for 8 days at 28 ± 2 °C. P-solubilization zones were observed, and the Phosphorus Solubilizing Index (PSI) was calculated using the following formula (Kumar and Narula, 1999).


$$Phosphorus\;Solubilizing\;Index\;\left(PSI\right)\;=\frac{Colony\;diameter\;+\;Halozone\;diameter}{Colony\;diameter}$$

IAA production was measured using the method of Gordon and Weber (1951). Bacterial cultures were grown in nutrient broth and fungus in potato dextrose broth with 5 mg mL^-1^ L-tryptophan, incubated for 4–5 days at 28 ± 2 °C. To estimate IAA, 2–3 drops of orthophosphoric acid and 4 mL of Salkowski’s reagent were added to the microbial supernatant, followed by incubation in the dark for 25 minutes. Pink color formation indicated IAA production, and absorbance was measured at 530 nm. Siderophore production was evaluated using the method outlined by Schwyn and Neilands (1987).

### 2.3 Molecular identification of microbes

Five bacterial strains used in this study were previously characterized (Bhattacharya et al., 2019; Mishra et al., 2018; Chauhan et al., 2022). Genomic DNA of B3 and B5 was extracted following García de Salamone et al. (2010), and the 16S rRNA region was amplified using universal primers (Forward 8F: 5′-AGAGTTTGATCCTGGCTCAG-3′; Reverse 1392R: 5′-ACGGGCGGTGTGTAC-3′) (Dastager et al., 2009). The gene was amplified in a 20 µL reaction mixture containing 40 ng genomic DNA, dNTP mix (10 mM each), primers (20 pmol each), PCR buffer (1X), and 1 U Taq DNA polymerase. Amplification was done in a Genei TM Techne TC-5000 PCR with the following cycle: denaturation at 94˚C for 10 min (1 cycle), followed by 30 cycles of denaturation at 94˚C, annealing (50˚C), and 1 min extension (72˚C), and 1 cycle of final extension at 72˚C for 10 min. The PCR product was purified and sequenced using the Sanger sequencing method. The nucleotide sequences were matched with the NCBI GenBank database using online tools (http://www.ncbi.nlm.nih.gov/BLAST/). A phylogenetic tree was constructed using MEGA 10.0 software according to Kumar et al. (2018) to determine molecular relationships, and sequence data were submitted to NCBI for accession numbers.

### 2.4 Compatibility of microbes with soybean at different S concentrations under greenhouse conditions

#### 2.4.1 Experimental setup

The greenhouse experiment was conducted at the CSIR-National Botanical Research Institute, Lucknow, India. Soybean variety JS-335 was provided by Jawaharlal Nehru Krishi Vishwa Vidyalaya, Jabalpur, India. The plants were maintained at 30 ± 2 °C with a 12-h photoperiod in Soilrite media with a modified Hoagland solution having varying concentrations of Sulfur. The experiment was performed under aseptic, non-field conditions.

#### 2.4.2 Biopriming of soybean seeds

Soybean seeds were surface sterilized with 1% sodium hypochlorite for 2 minutes, followed by 90% and 70% ethanol washes for 2 minutes each, and then rinsed with autoclaved distilled water (3–4 times). The seeds were bio-primed using B3 and B5 (1 × 10^9^ cells mL^−1^) (Bhattacharya et al., 2019; Mahmood et al., 2016). and TP-5 (1 × 10^8^ cells mL^−1^) (Singh et al., 2019) for 1 hour, then air-dried in sterile conditions. Bio-primed seeds were sown in Soilrite with varying S concentrations (100% S, 50% S, 25% S, SD, and CN) amended with Hoagland solution. The composition of Hoagland nutrient solution was 4 mM potassium nitrate (KNO_3_); 4 mM calcium nitrate Ca (NO_3_)_2_; 2 mM magnesium sulphate (MgSO_4_); 1.33 mM monosodium phosphate (NaH_2_PO_4_); 0.33 mM hydrogen borate (HBO_3_); 0.1 mM ferric ethylene di amine tetra acetic acid (Fe EDTA); 10 mM manganese sulphate (MnSO_4_); 1 mM copper sulphate (CuSO_4_); 1 mM zinc sulphate (ZnSO_4_); 0.1 mM sodium molybdate (Na_2_MoO_4_). Sulfur was applied in the form of magnesium sulphate (MgSO_4_), and magnesium was applied as magnesium chloride (MgCl_2_) in an S-deficient condition. Under S deprivation, MgSO_4_, MnSO_4_, ZnSO_4_, CuSO_4_ were respectively replaced by the same concentration of CuCl_2_, MgCl_2_, MnCl_2_, and ZnCl_2_ (Réthoré et al., 2020). Details of the treatments used in the experiments are given in below.

C+ SD: Control untreated with Sulfur deficient concentration; B3+SD: B3 treated plant with Sulfur deficient concentration; B5+SD: B5 treated plant with Sulfur deficient concentration; TP-5+SD: *Trichoderma* treated plant with Sulfur deficient concentration; C + 0%: Control; B3 + 0%: B3 treated plant with distilled water; B5 + 0%: B5 treated plant with distilled water; TP-5 + 0%: *Trichoderma* treated plant with distilled water; C + 25%S: Control untreated with 25% Sulfur concentration; B3 + 25%S: B3 treated plant with 25% Sulfur concentration; B5 + 25%S: B5 treated plant with 25% Sulfur concentration; TP-5 + 25%S: *Trichoderma* treated plant with 25% Sulfur concentration; C + 50%S: Control untreated with 50% Sulfur concentration; B3 + 50%S: B3 treated plant with 50% Sulfur concentration; B5 + 50%S: B5 treated plant with 50% Sulfur concentration; TP-5 + 50%S: *Trichoderma* treated plant with 50% Sulfur concentration; C + 100%S: Control untreated with 100% Sulfur concentration; B3 + 100%S: B3 treated plant with 100% Sulfur concentration; B5 + 100%S: B5 treated plant with 100% Sulfur concentration; TP-5 + 100%S: *Trichoderma* treated plant with 100% Sulfur concentration Modified concentration of S was used in this study:


$$100\%\text{S}=2{\text{mLMgSO}}_{4}$$

$$50\%\text{S}=1{\text{mL MgSO}}_{4}+1{\text{mL MgCl}}_{2}$$

$$25\%\text{S}=0.5{\text{mL MgSO}}_{4}+1.5{\text{mL MgCl}}_{2}$$

$$\text{SD}=\text{Deficient Sulfur}+2{\text{mL MgCl}}_{2}$$

C=Distilled water

Approximately 600 g of Soilrite with different S concentrations was added to each 4×4-inch micro pot, in which four soybean seeds were sown. After 45 days of treatment, all experiments were conducted, and the crop was harvested after 90 days. Upon reaching maturity, physiological characteristics such as root length, shoot length, number of pods, fresh weight, and dry weight were recorded (Singh et al., 2019). Soybean plant was treated with sulfur levels at SD, 0%, 25%, 50%, and 100%. Five treatments were accompanied by 3 microbial inoculations. Each treatment involved three biological replicates. Three biological replicates were prepared by pooling samples collected from three plants that received the same level of treatment. This implies that in each case, nine plants received the same treatment for physiological, biochemical, and molecular analyses.

### 2.5 Estimation of biochemical and defense responses in soybean

#### 2.5.1 Biochemical responses

The total chlorophyll and carotenoid content of the plants was determined using the method described by Arnon (1949). In brief, 100 mg of leaf tissue was homogenized in 2 mL of chilled 80% acetone. The homogenate was then centrifuged at 11,000 rpm at 4 °C for 5 minutes, and the supernatant was collected. Absorbance was recorded at 663, 645, 510, and 480 nm using a UV-visible spectrophotometer (Evolution, USA).

#### 2.5.2 Defense enzymes

Defense enzyme activity was measured in 45-day-old soybean plant tissues under various S concentrations. Root and shoot tissues were homogenized in 3 mL of chilled 100 mM potassium phosphate buffer (pH 7), containing 0.1 mM EDTA, 1% Polyvinyl pyrrolidone (PVP), and 0.3 mM Phenyl methyl sulfonyl fluoride (PMSF). The homogenate was then centrifuged at 11,000 rpm for 5 minutes at 4 °C, and the supernatant was stored at -80 °C until enzyme activity analysis. Superoxide dismutase (SOD) was estimated based on inhibition of photochemical reduction of Nitro Blue Tetrazolium (NBT) in the riboflavin/methionine system (Fridovich, 1975). The Catalase (CAT) activity was determined based on the rate of oxidation of H_2_O_2_ by following the method of Aebi (1974). The Phenol peroxidase (PPO) was estimated using 0.1 M catechol and expressed as U mg^-1^ Protein FW (Mohammadi and Kazemi, 2002). The Ascorbate peroxidase (APX) activity was determined according to Mishra et al. (2006).

### 2.6 Estimation of non-enzymatic stress parameters in soybean

#### 2.6.1 Lipid peroxidation

LPX activity was determined by measuring the content of Thiobarbituric acid reactive substances (TBARS) (Schmidt and Kunert, 1986). Briefly, 100 mg of fresh leaf tissue was ground in 3 mL of 0.1% TCA and centrifuged at 10,000 rpm for 5 minutes. The supernatant was mixed with 2 mL of 20% TCA containing 0.5% Thiobarbituric acid and incubated at 95 °C for 30 minutes, then cooled. Absorbance was measured at 532 and 600 nm, and TBARS content was expressed as nmol of TBARS per g of fresh weight.

#### 2.6.2 Total soluble sugar

Total soluble sugar content was determined in the root and shoot of soybean using the method of DuBois et al. (1956). Fresh tissue (100 mg) was homogenized in 2.5 mL of 80% methanol and incubated at 70 °C in a water bath for 1 hour. After incubation, 500 μL of the supernatant was mixed with 500 μL of 5% phenol and 5 mL of 95% H_2_SO_4_, then incubated in the dark for 15 minutes. The absorbance was measured at 490 nm.

### 2.7 Estimation of antioxidant activities

#### 2.7.1 Total flavonoid content

Total Flavonoid Content (TFC) was determined using the method described by Chang et al. (2002). Fresh leaves (50 mg) of the soybean plant were homogenized in 5 mL of methanol and shaken on an orbital shaker for 48 hours at room temperature. The solution was filtered and stored at 4 °C for later use. For the estimation of total flavonoid content, 1 mL methanolic solution was mixed with 0.2 mL 10% aluminum chloride solution in methanol (w/v), 0.2 mL 1 M potassium acetate solution, and 5.6 mL distilled water. The solution was incubated at room temperature for 30 minutes. The solution was then filtered, and the absorbance was recorded at 415 nm using a spectrophotometer.

Total flavonoid content was expressed as milligrams quercetin equivalent per gram fresh weight (QE mg/g FW).

#### 2.7.2 DPPH (2,2-diphenyl-1-picrylhydrazyl) free radical scavenging activities

DPPH free radical scavenging activity was performed according to the method described by Xu and Chang (2007). The fresh leaves (500 mg) of the soybean plant were homogenized in 10 mL of methanol and incubated in the dark with continuous shaking for 12 hours. The homogenate was centrifuged at 10,000 rpm for 5 minutes. The supernatant was collected and stored at 4 °C and used for the DPPH and ABTS assays. For the DPPH assay, the same volume of the extract and 0.1% methanolic solution of DPPH was mixed and incubated in the dark for 30 minutes. The absorbance was measured at 510 nm using the methanol-DPPH solution as the control. The formula determined the percentage of DPPH free radical scavenging activity:

DPPH radical scavenging activity (%) = (*Ac*−*At*)/*Ac*×100.

Where Ac- absorbance of DPPH; at -absorbance of DPPH with the sample.

#### 2.7.3 ABTS free radical scavenging activities

Measurement of the ABTS [2,2-azino-bis(3-ethylbenzothiazoline-6-sulfonic acid)] free radicals scavenging activity was carried out according to the method described by Lee et al. (2010). The ABTS free radicals were produced by reacting 7 mM ABTS solution with 2.5 mM potassium persulfate solution in the dark at room temperature for 12–16 hours. Before using the solution, it was diluted with 5 mL of methanol to adjust the absorbance to 0.70 ± 0.02 at 734 nm. To determine the free radical scavenging activity, 20 µL of the sample solution was mixed with 180 µL of the diluted solution containing the free radicals. After the reaction, the solution was read at 734 nm. Scavenging capacity was calculated using the formula.

ABTS radical scavenging activity (%) = (*Ac*−*At*)/*Ac*×100.

Where, Ac- absorbance of ABTS radical solution; at- absorbance of a mixture of ABTS radical and sample solution.

### 2.8 Phenylalanine ammonium lyase and tyrosine ammonium lyase

The activity of PAL and TAL enzymes was determined by the method of Beaudoin-Eagan and Thorpe (1985). The optimal pH for the activity of both enzymes was 8 for PAL and 8.5 for TAL. The reaction mixture for the enzymes was 0.5 mL of the enzyme extract, 150 mM of L-phenylalanine or L-tyrosine, and 3 mL of the extraction buffer. The reaction was carried out for 30 minutes at 30 °C for TAL and 40 °C for PAL. The activity of the enzymes was measured by the rate of Trans-cinnamic acid synthesis for PAL, measured at 290 nm, while the activity of TAL was measured by the rate of p-coumaric acid synthesis, measured at 330 nm.

### 2.9 Estimation of glutathione and S-containing amino acids

Plant leaves (500 mg) were frozen, homogenized in 0.1 M sodium phosphate buffer (pH 8.0) with 25% meta-phosphoric acid, and centrifuged at 13,000 rpm for 20 minutes at 4°C. GSH levels were determined fluorometrically in the supernatant after 15 minutes of incubation with o-phthaldialdehyde (OPT). Fluorescence intensity was measured at 420 nm (excitation at 350 nm) using a Hitachi F-7000 fluorescence spectrophotometer (Hissin and Hilf, 1976).

Sulfur-containing amino acid cysteine was estimated in soybean seeds through a spectrophotometer using a reaction with an acid ninhydrin reagent (Gaitonde, 1947), and methionine content in grain samples was estimated by Horn et al. (1946).

### 2.10 Estimation of macro and micronutrients in the soybean shoot

Total nitrogen content in soybean shoots was determined using the Kjeldahl method (Nelson and Sommers, 1973). Phosphorus content was measured by the method of John (1970), and S content was estimated using the method of Williams and Steinbergs (1959). Potassium (K) and calcium (Ca) concentrations were analyzed via flame photometry (CL 378 - ELICO) (Bisht and Chauhan, 2020). For microelements (Mn, Fe, Mo, Cu, Zn, B), 500 mg of tissue was digested in a microwave system using a mixture of 70% HNO_3_, 30% H_2_O_2_, and 48% HF. The digested solution was filtered, diluted, and analyzed by Inductively Coupled Plasma Mass Spectrometry (ICP-MS) (Thermo TQ) (Singh et al., 2015).

### 2.11 Scanning electron microscopy of soybean root

Root transverse sections were sliced using razor blades and fixed for 1 hour in 2.5% glutaraldehyde in 0.1 M sodium cacodylate buffer (v v^-1^). The fixed tissues were then treated with 1% osmium tetroxide for 40 minutes, followed by dehydration through increasing ethanol concentrations (10–100%). After drying in a critical point dryer, the sections were gold-coated for 60 seconds and examined using a scanning electron microscope (Quanta 250, FEI, USA) (Mishra and Nautiyal, 2018). Scale bars were generated and calibrated using the SEM software.

### 2.12 Gene expression profiling of soybean leaves by qRT-PCR analysis

Total RNA was extracted from soybean leaves using the Spectrum™ Plant Total RNA Kit (Sigma-Aldrich, USA) following the manufacturer’s guidelines. The extracted RNA was treated with DNase to remove traces of genomic DNA contamination. For the qRT-PCR experiment, the cDNA was synthesized from 1 µg of total RNA using the Maxima H Minus First-Strand cDNA Synthesis Kit (Thermo Scientific, USA). The synthesized cDNA was diluted (1:10) and used as a template to perform the qRT-PCR experiment. For the qRT-PCR experiment, the reaction was carried out using a 10 µL reaction volume containing QuantiTect™ SYBR^®^ Green PCR Master Mix (Agilent Technologies, USA), diluted cDNA, and gene-specific primers. The thermal conditions were: initial denaturation at 94 °C for 5 min; 35 cycles of denaturation at 94 °C for 30 s; annealing at 50-58 °C for 30 s; and extension at 72 °C for 30 s. Eight genes involved in S metabolism (*SULTR1, SULTR2, SAT2, ATPS, SIR, NIR, DES, GR*) were analyzed, with soybean Actin used as a reference. Gene primers were designed using IDT Primer Quest software. Relative gene expression was quantified using the 2−ΔΔCt method (Livak and Schmittgen, 2001). A heat map was generated with GraphPad Prism 8. Primers used for qRT-PCR analysis are listed in **Supplementary Table 2**.

### 2.13 Statistical analysis

Statistical analysis was performed using SPSS version 18.0 (SPSS Japan). Two-way ANOVA and ‘Tukey’s test (HSD) (p ≤ 0.05) were used to analyze physical parameters and enzyme assay data. Both *in-vitro* and *in-vivo* experiments were repeated in triplicate to validate the results.

## 3 Results

### 3.1 Sulfur solubilization

**Figure 1:**
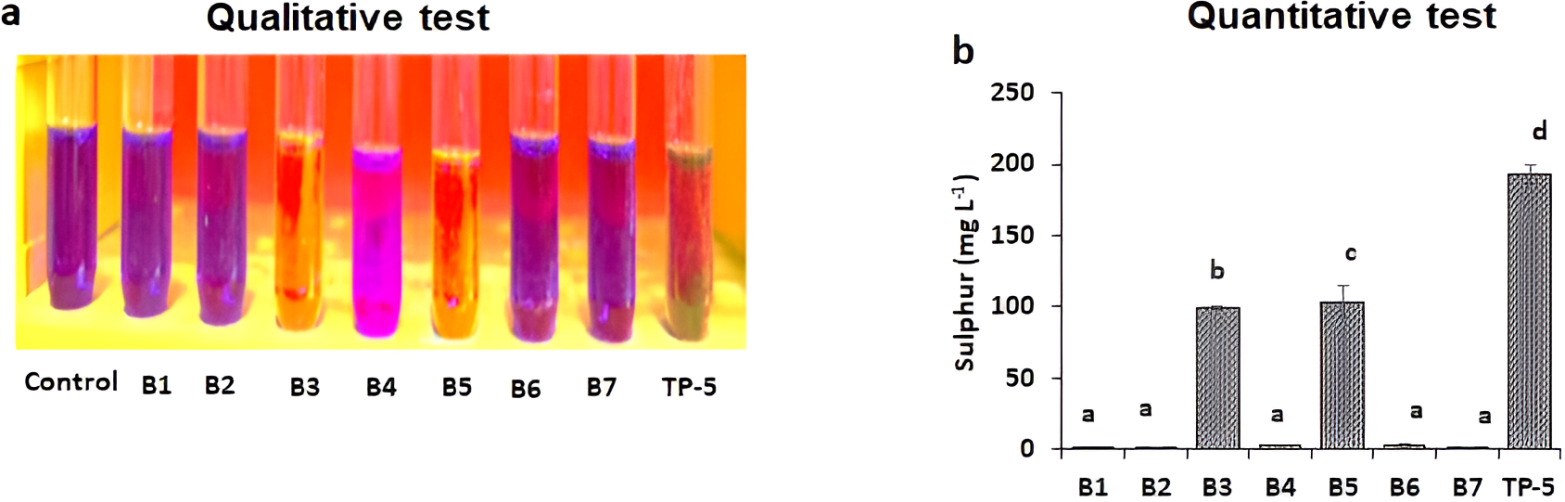
*In vitro* estimation of S oxidizing microbes **(A)** Sulfur oxidizing test **(B)** Quantitative estimation of Sulfur. Bars represent standard errors from three replicates. The different letters indicate significant differences (p ≤ 0.05) according to ‘Tukey’s test (HSD).

The Sulfur-solubilizing activity was found to be higher in *Brevibacillus brevis* (B3), *Bacillus* sp. (B5), and *Trichoderma reesei* (TP-5) than in the other isolates (**Figure 1a**). The oxidation of thiosulphate was indicated by the color change of the medium from purple to yellow. The quantitative analysis revealed significantly higher Sulfur production in B3 (98.99 mg L^−1^), B5 (103.69 mg L^−1^), and TP-5 (192.61 mg L^−1^) than in the other isolates (**Figure 1b**).

### 3.2 Plant growth- promoting activity

All microbial strains exhibited plant growth-promoting activities, including P-solubilization, siderophore production, and indole-3-acetic acid (IAA) production. TP-5 (2.83 PSI), B5 (2.32 PSI), and B3 (2.35 PSI) exhibited the higher P-solubilization activity (**Supplementary Figure 1**; **Table 2**). TP-5 also showed the higher IAA production (30.56 µg mL^−1^), followed by B2 (25.6 µg mL^−1^), while B4 had the lowest production (10.25 µg mL^−1^) (**Table 2**). B3, B5, and TP-5 exhibited higher S solubilization and plant growth-promoting activity compared to the other strains (B1, B2, B4, B6, and B7). Hence, these strains were chosen for further study.

**Table 2:** *In vitro* characterization of plant growth-promoting activity of microbes.

Microbial strains	Plant growth-promoting activity			
	Qualitative estimation		Quantitative estimation	
	Siderophore production	Phosphorus solubilization	IAA production (µg mL-1)	P-solubilizing index (PSI)
B1	+	+	20.56 ± 0.23	2.29 ± 0.25
B2	+	+	25.56 ± 0.52	2.23 ± 0.5
B3	+	+	12.25 ± 0.05	2.35 ± 0.02
B4	+	+	10.28 ± 0.05	1.00 ± 0.15
B5	+	+	15.36 ± 0.15	2.32 ± 0.04
B 6	+	+	18.25 ± 0.15	2.21 ± 0.24
B7	+	+	16.59 ± 0.52	1.66 ± 0.06
TP-5	+	+	30.56 ± 0.18	2.83 ± 0.12

Values are mean of three replicates with ± standard error (SE) are indicated. ‘+’ presented activity.

All bacterial isolates possessed plant growth-promoting traits, such as phosphate solubilization, siderophore production, and indole-3-acetic acid (IAA) production. The higher phosphate solubilization index (PSI) of 2.83 was recorded by TP-5, followed by B3 (2.35) and B5 (2.32) (**Supplementary Figure 1**; **Table 2**). The maximum IAA production was observed in TP-5 (30.56 µg mL^−1^), followed by B2 (25.6 µg mL^−1^), and the lowest production was recorded by B4 (10.25 µg mL^−1^).

Based on the highest Sulfur solubilization and plant growth-promoting potential, B3, B5, and TP-5 were selected for subsequent greenhouse evaluation.

### 3.3 Molecular characterization of microbes

The 16S rRNA gene sequences of B3 and B5 were amplified and sequenced. The BLAST result showed 98.97% similarity of B3 to *Brevibacillus brevis* and 100% similarity of B5 to *Bacillus* sp. The sequences were submitted to GenBank with accession numbers ON912007 and ON912056 (**Supplementary Figure 2**).

### 3.4 Compatibility of microbes with soybean at different S concentrations under greenhouse conditions

#### 3.4.1 Physiological parameters

Under greenhouse conditions (**Supplementary Figure 3**), microbial inoculation resulted in significant improvement of growth parameters of soybean plants with varying concentrations of Sulfur. TP-5-treated plants showed the maximum degree of enhancement in growth parameters at lower concentrations of Sulfur. At 25% S, TP-5 treatment resulted in 3.88-fold, 1.89-fold, 5.5-fold, and 8.36-fold increases in shoot length, root length, fresh weight, and dry weight, respectively, compared to the control C + 25%S (**Supplementary Table 1**).

Germination of seeds was significantly higher in TP-5 + 25%S and TP-5 + 50%S treatments (3.0-fold and 2.80-fold increases, respectively). Number and weight of pods were also significantly increased in TP-5 + 25%S and TP-5 + 100%S treatments compared to the control. Improvement in pod quality was also observed.

B3 increased growth at 25% and 50% S concentrations, whereas B5 showed relatively lower efficacy.

#### 3.4.2 Biochemical parameters

The contents of chlorophyll and carotenoids were highly variable among treatments (**Supplementary Figures 4a, b**). The highest content of chlorophyll a was measured in TP-5 + 25%S (1.09 mg g^-1^ FW), while the lowest was in B3 (0.26 mg g^-1^ FW). The highest content of chlorophyll b was in TP-5 + 100%S (1.7 mg g^-1^ FW), while the lowest was in B3 (0.04 mg g^-1^ FW).

Total chlorophyll (2.75 mg g^-1^ FW) and carotenoid (4.6 mg g^-1^ FW) contents were significantly higher in TP-5 + 25%S-treated plants compared to other treatments.

### 3.5 Stress-ameliorating enzymes

**Figure 2:**
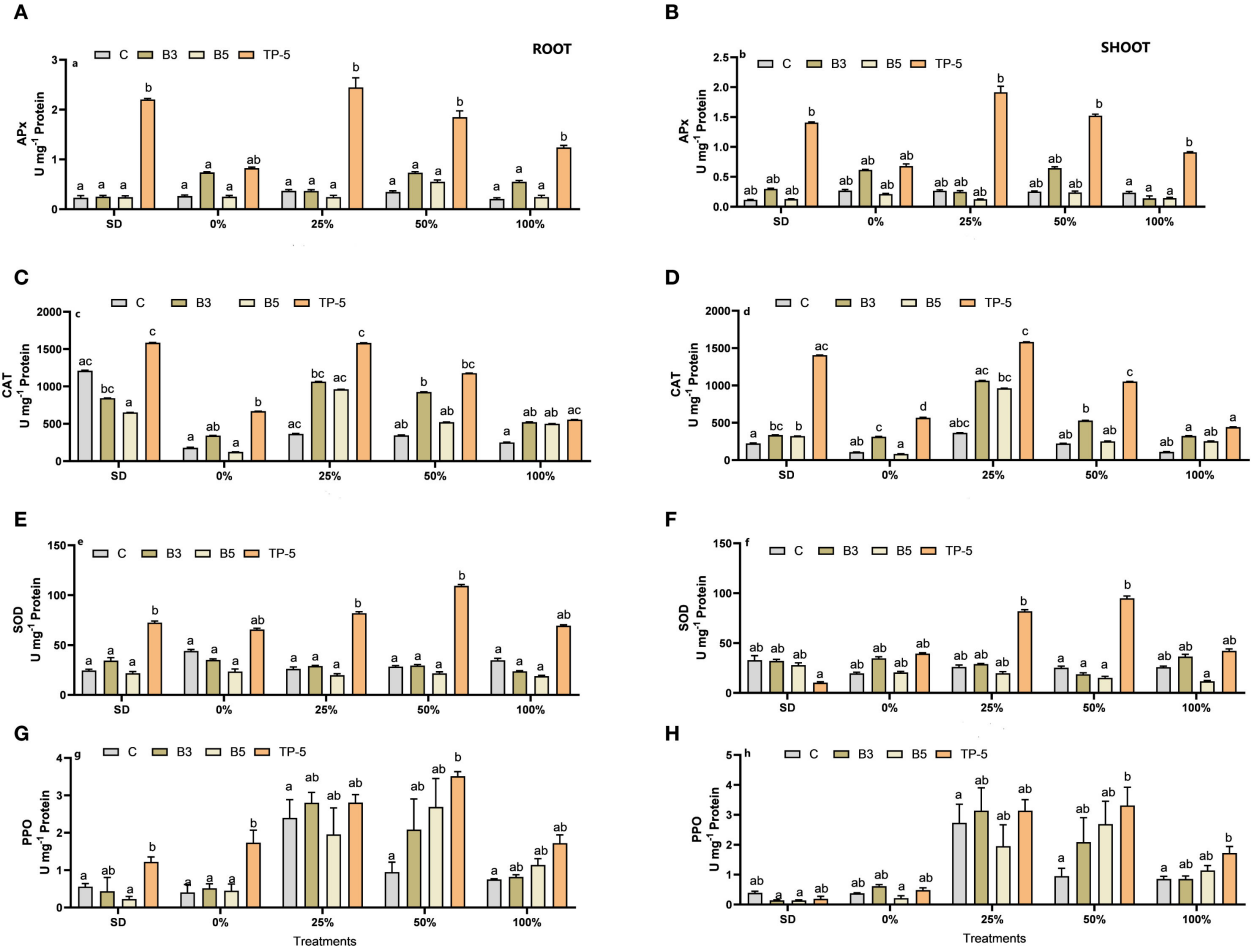
Effect of defense-related enzymes **(A, B)** APX, **(C, D)** CAT, **(E, F)** SOD, and **(G, H)** PPO on soybean root and shoot grown in different S concentrations in the absence and presence of different microbes (B3, B5, and TP-5) treatments. Bars represent standard errors from three replicates. The different letters indicate significant differences (p ≤ 0.05) according to ‘Tukey’s test (HSD).

The activity of antioxidant enzymes was significantly affected by microbial inoculation and Sulfur concentration (**Figure 2**). The highest APX activity was recorded in TP-5 + 25%S (2.01 U mg^−1^ protein in roots and 1.23 U mg^−1^ protein in shoots), while the lowest activity was found in B5 + 25%S and B5+SD. (**Figures 2a, b**).

The highest CAT activity was found in TP-5 + 25%S and TP-5 + 50%S (1583.71 U mg^−1^ protein in roots and 1155.63 U mg^−1^ protein in shoots), while the lowest was recorded in B5-treated plants. (**Figures 2c, d**).

Similarly, the highest SOD activity was recorded in TP-5 + 50%S (108.11 U mg^−1^ protein in roots and 100.21 U mg^−1^ protein in shoots), while the lowest was found in B5 + 100%S. (**Figures 2e, f**).

The highest PPO activity was found in TP-5 + 50%S roots (3.43 U mg^−1^ protein) and B3 + 50%S shoots (2.02 U mg^−1^ protein). Conversely, B5+SD treatment resulted in the lowest PPO activity in shoots (0.12 U mg^−1^ protein) (**Figures 2g, h**).

### 3.6 Assessment of non-enzymatic parameters

#### 3.6.1 Total soluble sugar

The minimum total soluble sugar (TSS) content in the root and shoot of B5 + 25%S soybean is 5.23 µg g^-1^ FW and 2.86 µg g^-1^ FW, respectively. The maximum concentration of total soluble sugar was recorded in C + 25%S-treated roots and shoots, measuring 12.32 µg g^-1^ FW and 7.26 µg g^-1^ FW, respectively (**Supplementary Figures 5a, b**).

#### 3.6.2 Lipid peroxidation

Malondialdehyde (MDA) content was formed due to lipid peroxidation activity, which was maximum in the root and shoot of untreated plant C+SD (18.83 nmole of TBARS g^-1^ FW and nmole of TBARS g^-1^ FW). Minimum MDA content was found in TP-5 + 25%S treated root and shoot of soybean (7.28 nmole of TBRAS g^-1^ FW and 1.25 nmole of TBARS g^-1^ FW) (**Supplementary Figures 5c, d**).

### 3.7 Antioxidant activity response in soybean

The total flavonoid content was higher in TP-5 + 50%S and TP-5 + 25%S treated plants compared to B3 + 25%S and their controls (**Table 3**). Antioxidant activity, assessed by DPPH and ABTS assays, was also greater in TP-5 + 50%S (70.70% and 23.98%) and TP-5 + 25%S (77.99% and 20.59%), with the lower activity in B3+SD (4.85%). PAL activity peaked in TP-5 + 100%S (121.07 μmol TCA mg^−1^ protein), while TAL activity was higher in TP-5 + 25%S (4.56 μmol mg^−1^ protein) (**Table 3**).

**Table 3:** Effect of microbes on antioxidant activity in soybean shoot under Sulphur deficiency with different sulphur concentrations.

S. no.	Treatments	Total flavonoids (QE g^-1^)	DPPH (µg mL^-1^)	ABTS (µg mL^-1^)	PAL (μ mole TCA mg^-1^protein)	TAL (μ mole mg^-1^ protein)
1.	C+SD	3.16 ± 0.16^bcd^	40.62 ± 0.36 ^b^	12.36 ± 0.2^cd^	69.26 ± 0.51^a^	1.71 ± 0.047^bc^
2.	B3+ SD	2.52 ± 0.73^ab^	42.7 ± 0.3^b^	4.85 ± 0.3^a^	73.66 ± 0.26^a^	2.81 ± 0.25^ab^
3.	TP-5+ SD	3.35 ± 0.11^cd^	64.7 ± 2.44^d^	11.9 ± 0.35^cd^	89.26 ± 1.85^c^	3.85 ± 0.10^a^
4.	C+0%	2.39 ± 0.45^a^	62.46 ± 0.41^d^	12.92 ± 0.46^d^	40.19 ± 0.89^ab^	1.65 ± 0.15^a^
5.	B3 + 0%	2.25 ± 0.1^a^	53.76 ± 1.04^c^	12.00 ± 0.38^cd^	49.49 ± 1.50^bc^	2.33 ± 0.29^ab^
6.	TP-5 + 0%	3.28 ± 0.073^cd^	62.1 ± 1.41^d^	13.96 ± 0.35^cd^	96.99 ± 2.50^d^	2.82 ± 0.15^a^
7.	C+ 25%S	2.59 ± 0.5^ab^	44.58 ± 1.19^e^	10.42 ± 0.30^c^	71.56 ± 0.35^ab^	1.99 ± 0.028^ab^
8.	B3 + 25%S	2.56 ± 0.12^ab^	51.26 ± 0.6^b^	6.88 ± 0.3^ab^	85.14 ± 0.35^ab^	2.96 ± 0.12^a^
9.	TP-5 + 25%S	3.51 ± 0.34^d^	77.99 ± 1.37^c^	20.70 ± 0.2^e^	115.22 ± 1.79^c^	4.56 ± 0.006^a^
10.	C+ 50%S	2.41 ± 0.2^a^	34.63 ± 1.2^e^	8.07 ± 0.4^b^	72.60 ± 0.10^a^	2.753 ± 0.047^bc^
11.	B3 + 50%S	3.14 ± 0.37^bcd^	42.3 ± 0.58^a^	7.55 ± 0.33^b^	72.74 ± 0.86^b^	2.67 ± 0.22^ab^
12.	TP-5 + 50%S	3.27 ± 0.39^cd^	70.7 ± 0.30^b^	23.98 ± 0.37^e^	87.49 ± 0.64^ab^	4.23 ± 0.027^ab^
13.	C+ 100%S	2.72 ± 0.32^b^	61.25 ± 0.87^d^	12.02 ± 0.89^cd^	90.18 ± 1.39^c^	2.07 ± 0.09^a^
14.	B3 + 100%S	2.0 ± 0.61^a^	53.26 ± 0.60^e^	17.14 ± 0.50^d^	93.24 ± 0.085^b^	2.33 ± 0.13^a^
15.	TP-5 + 100%S	3.3 ± 0.18^cd^	73.1 ± 0.43^c^	17.11 ± 0.4^d^	121.07 ± 1.08^bc^	3.42 ± 0.027^a^

Values are means of three replicates ± standard error (SE) indicated. The superscripted lowercase letters in **Tables 3** and **4** indicate statistically significant differences among groups; values with different letters differ significantly, whereas values sharing the same letter are not significantly different.

### 3.7 Glutathione and S-containing amino acids content

**Figure 3:**
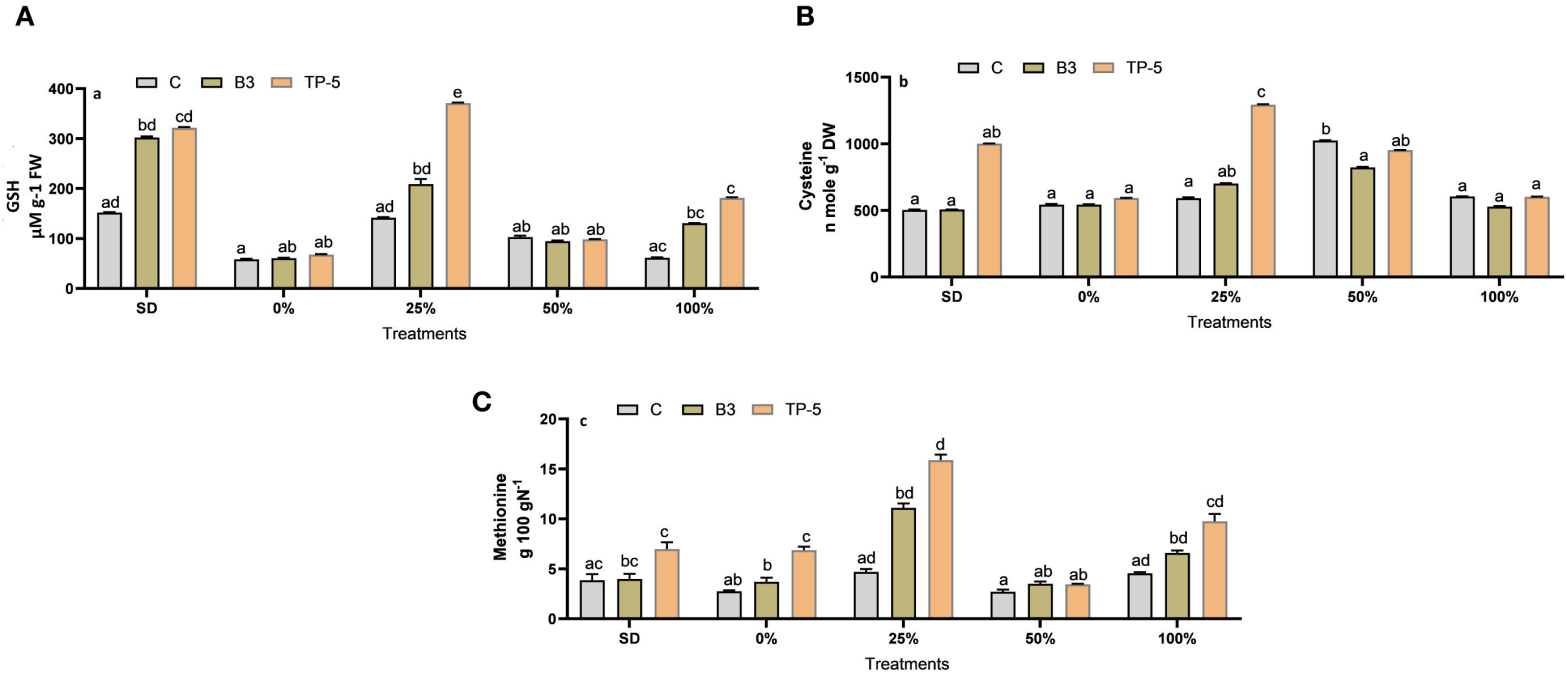
Effect of different S concentrations on **(A)** GSH of soybean shoots and Sulfur-related amino acid **(B)** Cysteine and **(C)** Methionine, of soybean seeds in the absence and presence of different microbes (B3 and TP-5) treatments. Bars represent standard errors from three replicates. The different letters indicate significant differences (p ≤ 0.05) according to ‘Tukey’s test (HSD).

Higher glutathione levels were found in TP-5 + 25%S (372.81 µM g^−1^ FW) and TP-5+SD (333.84 µM g^−1^ FW) treated soybean leaves, with the lower in C + 0% (61.92 µM g^−1^ FW) (**Figure 3a**). TP-5 + 25%S soybean seeds showed significant increases in cysteine (83%) and methionine (44.59%) compared to B3 + 25%S (**Figures 3b, c**). The lower cysteine and methionine levels were observed in C+SD (299.09 n mol g^−1^ DW) and C + 0% (3.37 g 100gN^−1^).

### 3.8 Macro and micronutrient analysis

The macro-nutrients nitrogen (N), phosphorus (P), Sulfur (S), potassium (K), and calcium (Ca) were reduced by 0.50 to 2.9-fold in the control compared to TP-5 treated plants under different S concentrations. TP-5-treated plants exhibited higher nutrient absorption at 50%S (1.13, 2.63, 2.85, 3.38, and 3.4-fold increase) and 25%S (3.71, 6.8, 4.17, 4.17, and 2.69-fold increase) compared to controls (**Table 4**). For micronutrients, boron (B), iron (Fe), nickel (Ni), copper (Cu), and zinc (Zn) showed increases in TP-5 + 25%S (2.69, 1.31, 1.8, 1.8, and 1.3-fold) and TP-5 + 50%S (1.25, 1.64, 2.2, 1.6, and 4.34-fold) treatments, while no significant changes were observed in TP-5 and TP-5 + 100%S treatments (**Table 4**).

**Table 4:** Nutrient uptake (Macro and Micronutrients) fluxes of soybean plants grown in different Sulphur concentration in absence and presence of TP-5 through ICP-MS.

S.N.	Treatments	Macronutrients					Micronutrients (mg g-1)				
		N (g kg^-1^)	P (mg kg^-1^)	S (mg kg^-1^)	K (mg kg^-1^)	Ca (mg kg^-1^)	B (mg kg^-1^)	Fe (mg kg^-1^)	Ni (mg kg^-1^)	Cu (mg kg^-1^)	Zn (mg kg^-1^)
1.	C+SD	2.3 ± 0.13a	0.6 ± 0.11^a^	6.7 ± 0.17^bc^	17.3 ± 0.14^b^	21.3 ± 0.23^c^	23.0 ± 0.29^a^	95.3 ± 12.36^cd^	0.7 ± 0.51^a^	0.7 ± 0.08^a^	11.75 ± 0.58^b^
2.	TP-5+SD	5.8 ± 1.65^bc^	1.9 ± 0.04^bc^	17.9 ± 0.24^cd^	51.7 ± 0.25^c^	41.4 ± 0.22^d^	27.9 ± 0.32^c^	131.2 ± 0.09^e^	1.6 ± 0.17^b^	2.05 ± 0.57^d^	15.76 ± 0.57^b^
3.	C+0%	4.0 ± 0.21^b^	0.2 ± 0.02^a^	1.9 ± 0.06^a^	8.6 ± 0.01^a^	6.9 ± 0.01^a^	21.4 ± 0.24^a^	33.4 ± 1.44^a^	1.4 ± 0.05^b^	1.0 ± 0.88^a^	16.16 ± 0.88^a^
4.	TP-5 + 0%	7.5 ± 0.12^bc^	1.8 ± 0.05^bc^	6.9 ± 0.51^bc^	58.1 ± 0.71^c^	31.7 ± 0.13^cd^	28.9 ± 0.83^c^	80.1 ± 0.13^bc^	1.5 ± 0.2^b^	1.5 ± 1.39^ab^	20.11 ± 1.39^bc^
5.	C+25%S	5.3 ± 1.02^b^	0.5 ± 0.07^a^	6.7 ± 0.08^bc^	16.9 ± 0.27^b^	23.9 ± 0.02^c^	37.9 ± 11.21^bc^	103.0 ± 3.25^c^	1.2 ± 0.02^b^	1.5 ± 0.08^ab^	15.72 ± 0.08^a^
6.	TP-5 + 25%S	19.7 ± 0.51^d^	3.4 ± 0.17^d^	28.7 ± 0.57^e^	92.3 ± 0.47^e^	62.7 ± 0.21^ef^	59.7 ± 0.60^d^	135.27.10^e^	2.2 ± 0.01^b^	2.7 ± 0.15^e^	20.1 ± 0.15^bc^
7.	C+50%S	13.71 ± 0.05^c^	1.1 ± 0.19^b^	9.1 ± 0.15^bc^	18.7 ± 0.56^b^	15.3 ± 0.21^b^	20.5 ± 0.27^a^	92.0 ± 0.19^c^	1.5 ± 0.09^b^	1.5 ± 0.42^ab^	23.99 ± 0.43^bc^
8.	TP-5 + 50%S	15.5 ± 0.21^c^	2.9 ± 0.40^cd^	26.1 ± 0.80^d^	61.1 ± 0.13^d^	52.2 ± 0.02^e^	25.7 ± 0.05^b^	151.5 ± 5.40^f^	3.3 ± 0.25^d^	2.4 ± 0.82^d^	100.94 ± 0.82^e^
9.	C+100%S	4.5 ± 0.23^b^	1.6 ± 0.02^bc^	4.2 ± 0.24^b^	15.5 ± 0.5b	11.1 ± 0.07^b^	21.1 ± 0.44^a^	50.2 ± 0.56^b^	0.9 ± 0.01^a^	1.7 ± 2.8^ab^	36.79 ± 2.81^c^
10.	TP5 + 100%S	15.0 ± 0.05^c^	2.5 ± 0.40^c^	13.4 ± 0.18^c^	70.9 ± 0.35^de^	62.4 ± 0.23^ef^	28.9 ± 0.67^c^	98.0 ± 0.1^cd^	1.3 ± 0.27^b^	1.8 ± 1.20^c^	67.16 ± 1.20^d^

Values are means of three replicates ± standard error (SE) indicated. The superscripted lowercase letters in **Tables3** and **4** indicate statistically significant differences among groups; values with different letters differ significantly, whereas values sharing the same letter are not significantly different.

### 3.9 Scanning electron microscopy of soybean root

**Figure 4:**
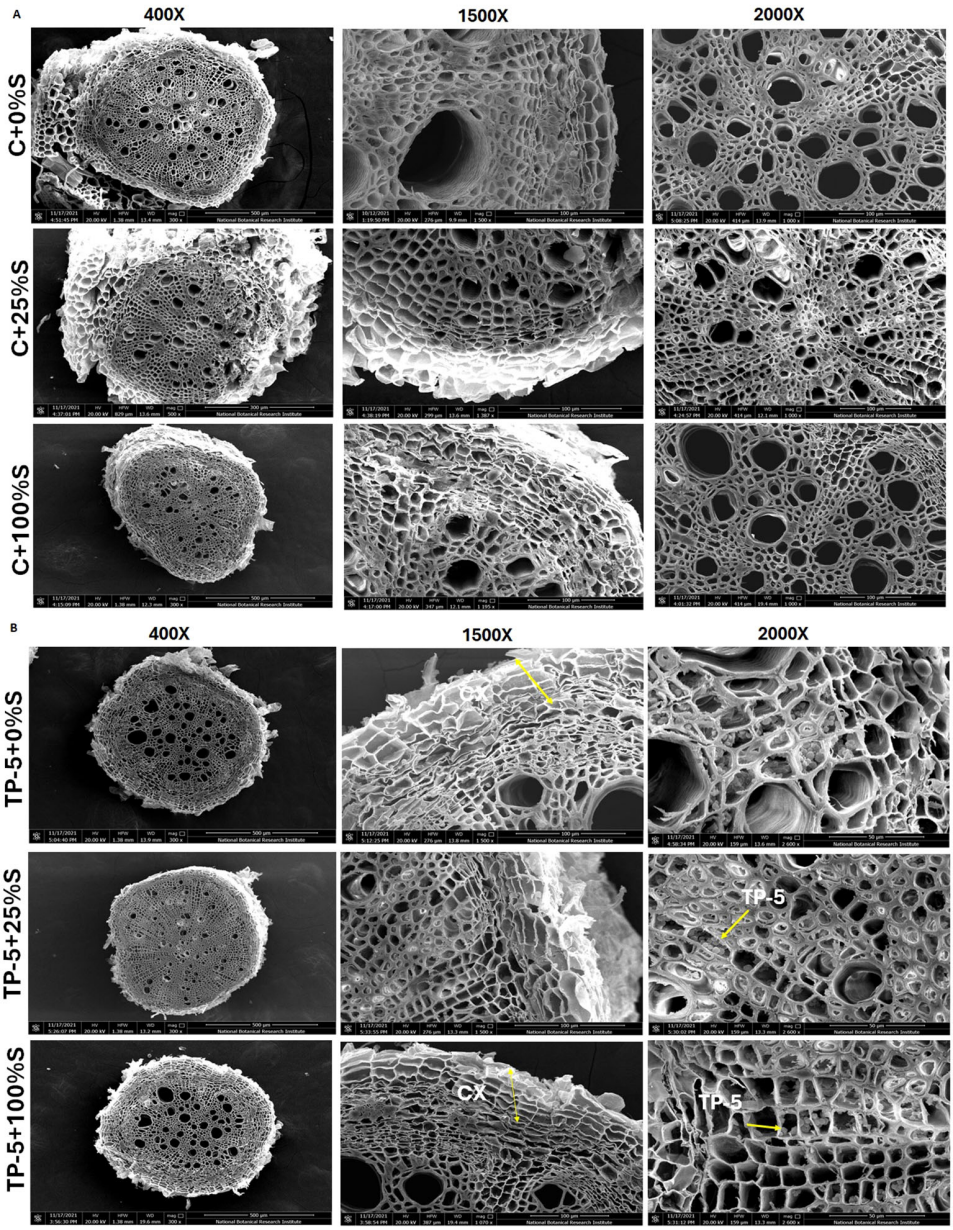
Scanning electron micrographs of soybean root control: **(A)** C + 0%, C + 25%S, C + 100%S, and **(B)** *Trichoderma reesei* treated; TP-5 + 0%, TP-5 + 25%S, TP-5 + 100%S under different S concentrations. (CX- Cortex; TP-5- *Trichoderma reesei*).

Soybean roots colonization by *Trichoderma reesei* (TP-5) could be seen through SEM results (**Figures 4a, b**). The fungal spores were found in the soybean roots. Soybean roots in the control group with lower sulfur content (C + 25%S) showed an irregular structure in both epidermal and cortical cells. However, the treated roots (TP-5 + 25%S) showed a more organized tissue architecture and apparent differences in metaxylem and sclerenchyma cell organization relative to the control treatments. These observations are qualitative in nature and provide supportive anatomical evidence of the effects associated with TP-5 inoculation.

### 3.10 Gene expression profiling of soybean by real-time PCR analysis

**Figure 5:**
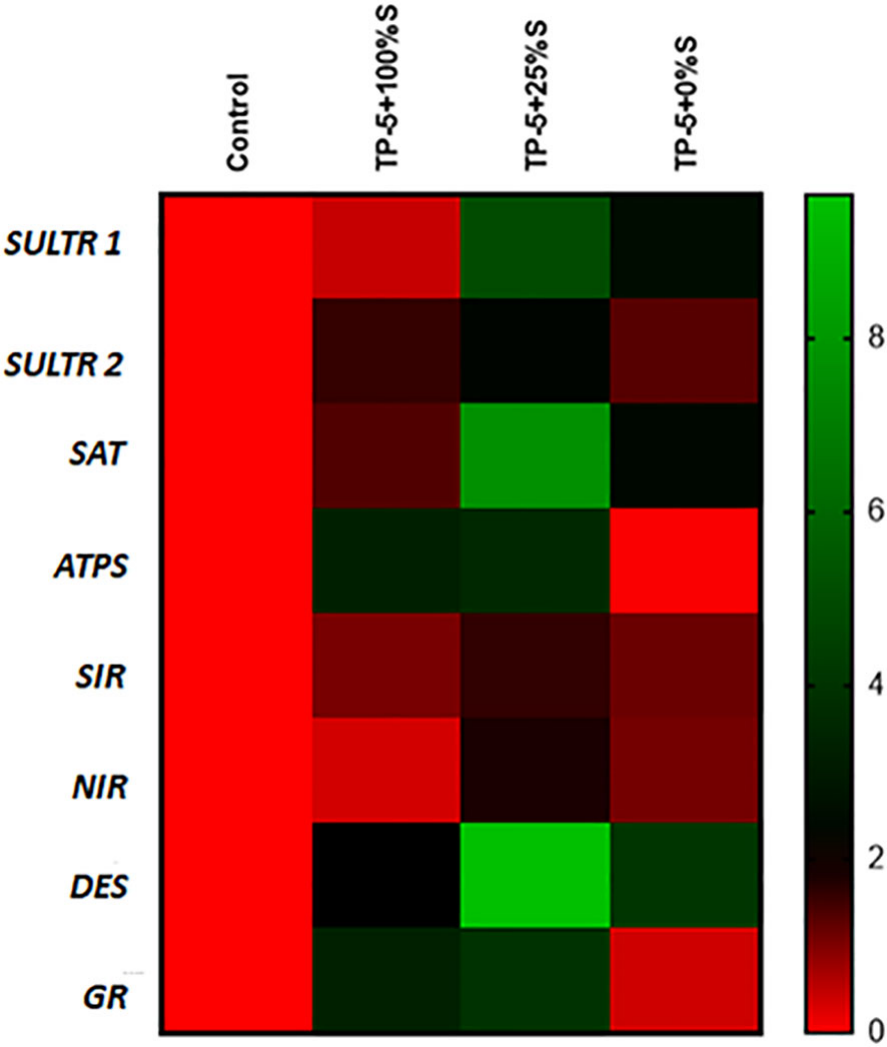
Differential expression of S metabolism-related genes in soybean inoculated with *Trichoderma reesei* at different concentrations of S. The heat map was generated from log2 fold-change values for treated samples compared with control plants. The color scale for log_2_ fold change values is shown at the right.

In the presence of *Trichoderma*, the expression of S metabolism-related genes was significantly upregulated in TP-5 + 25%S, with fold increases as follows: Sulfur transporter 1 (*SULTR1*) (4.9-fold), serine acetyltransferase (*SAT 2*) (7.7-fold), D-cysteine desulfhydrase (*DES*) (9.66-fold), glutathione reductase (*GR*) (3.9-fold), ATP Sulfuryase (*ATPS*) (3.59-fold), nitrite reductase (*NIR*) (1.79-fold), and sulfite reductase (*SIR*) (1.16-fold). *SULTR2* was upregulated in TP-5 + 100%S treatment compared to controls. However, the expression of these genes gradually decreased under TP-5 + 100%S treatment. Downregulation was observed for *SULTR1* (0.44-fold), *SAT 2* (1.36-fold), *DES* (2.0-fold), and *NIR* (0.34-fold) in TP-5 + 100%S. The expression levels of all S metabolism-related genes are shown in the heat map (**Figure 5**).

## 4 Discussion

A sulfur (S) deficiency constitutes one of the primary limitations to crop production due to soil depletion and present-day farming systems since S is a vital component in the formation of sulfurous amino acids, proteins, enzymes, and antioxidant molecules that play a key role in regulating photosynthesis and metabolism processes (Zandalinas et al., 2018). S directly affects photosynthesis and metabolism in plants due to a molecular constituent of cysteine and methionine, and a redox homeostasis regulator and chlorophyll synthesis regulator (De eBont et al., 2023). Consistent with earlier reports describing impaired and oxidative imbalance under S-deficient plants (Vidal et al., 2025; Hong et al., 2026), this study shows that *Trichoderma reesei* (TP-5) improves Sulfur acquisition and physiological performance of soybean, particularly under 25% and 50% sulfur supply treatments, which elicited the most favorable responses among the sulfur regimes evaluated.

*In vitro* assay confirmed that TP-5 possesses multiple plant growth-promoting traits such as Sulfur and phosphorus solubilization, IAA production, and siderophores secretion, which are similar to previous reports on beneficial *Trichoderma*-plant interaction (Vinale et al., 2008; Mishra et al., 2022; Singh et al., 2023). Notably, these traits were functionally translated under greenhouse conditions, where TP-5 inoculation enhanced plant biomass accumulation and Sulfur uptake under 25% and 50% sulfur supply treatments. These results indicate that microbial effectiveness is strongly influenced by plant nutrient status, supporting earlier observations that plant-microbe interactions are maximized under suboptimal nutrient availability (Jacoby et al., 2017; Wang et al., 2025).

The improved growth in TP-5 + 25%S and TP-5 + 50%S treatments was associated with the restoration of chlorophyll and carotenoid content, implying the maintenance of photosynthetic activity under nutrient stress. Sulfur deficiency is known to impair chloroplast function and pigment synthesis (Frydenvang et al., 2015), and the enhanced pigment retention observed in this study suggests improved metabolic integrity. This physiological recovery coincided with significantly increased activities of antioxidant enzymes (SOD, CAT, and APX) and reduced lipid peroxidation (lower MDA content. This finding suggests that antioxidant enzymes showed higher activities along with an increase in the DPPH radical scavenging activity, whereas the MDA level was decreased, which suggests that inoculation with TP-5 regulated the antioxidant defense system in response to deficiency in sulfur. Decreased MDA level also indicated reduced lipid peroxidation in TP-5-inoculated plants. Nonetheless, since ROS generation, H_2_O_2_ level, superoxide radicals, and membrane stability were not measured, such changes could be considered indicators of increased antioxidant potential but not improved stress tolerance yet (Noctor et al., 2012; Gulshan et al., 2022; Zandi and Schnug, 2022). Although moderate variability is observed, the consistency in the increased antioxidant enzyme activity in the biological replicates suggests consistency in the physiological response to the treatment with TP-5. The observed 77% enhancement in the radical scavenging activity is due to the substantial biological response to the treatment with TP-5.

Enhanced redox regulation was further supported by increased glutathione (GSH) content and the accumulation of cysteine and methionine in TP-5 treated plants. As cysteine serve as a rate-limiting precursor for the synthesis of GSH, improved Sulfur assimilation likely enhanced thiol metabolism, thus strengthening cellular redox buffering capacity (Kopriva et al., 2019). Such improvements in thiol-based antioxidant capacity due to stress and biotic stimulation have also been reported in legumes (Noctor et al., 2024), thus supporting the physiological significance of our findings.

Sulfur nutrition is closely integrated with the metabolism of other mineral nutrients, particularly nitrogen, and Sulfur deficiency often results in disruption of N–S balance (Narayan et al., 2023; Carciochi et al., 2017). In the current study, TP-5 inoculation under 25% and 50% S conditions significantly enhanced the accumulation of both macro and micronutrients compared to non-inoculated controls. The concurrent increase in nitrogen and Sulfur contents suggests partial restoration of nutrient homeostasis, which likely contributed to improved growth and seed quality under Sulfur-limited conditions. The low variability observed in the biological replicates suggests consistency in the performance of the microbes. The increase in the content of Sulfur (4.17-fold increase) in the TP-5-treated plants is not due to marginal statistical significance but rather to a substantial biological response. This is because the increase in the content of Sulfur is due to the enhanced nutrient mobilization efficiency in response to Sulfur deficiency. It is noteworthy that there was an increase in Cu and Ni content especially in the treatment of TP-5 + 25% S. Copper is a micromineral that participates in various enzymatic and redox reactions while nickel is needed only in small amounts as a component of urease. It is unclear whether these differences are a consequence of a change in nutrient uptake due to sulfur deficiency since no study on the reason behind their accumulation was done.

Qualitative anatomical studies of the roots using SEM provided additional insights to the physiological studies conducted in this study. Roots treated with TP-5 and grown on sulfur-deficient medium showed better organization of tissue compared to the control plants. Such alterations in root anatomy due to microbial interactions have also been observed in nutrient-deficient environments and are thought to contribute to improved uptake and transport of nutrients (El Amrani, 2023; Lopez et al., 2023). However, because quantification of the anatomical data was not done, the observations can only be taken as supportive evidence for the physiological results. Microbe-mediated root architectural plasticity has been observed in response to various nutrient deficiencies (Patel et al., 2025). Such anatomical changes could potentially promote improved nutrient transport, although hormonal regulation was not examined in this study.

At the molecular level, TP-5 inoculation under moderate S deficiency induced ATP sulfurylase (ATPS), the first committed enzyme of sulphate assimilation, consistent with increased Sulfur content and enhanced thiol accumulation. In contrast, sulfite reductase (SIR) expression exhibited comparatively moderate regulation, suggesting feedback control at downstream steps to maintain metabolic balance and prevent excessive sulphite accumulation (Zenda et al., 2021). Importantly, the transcription level of D-Cysteine Desulfhydrase (DES) increased considerably when TP-5 was used in plants with low amounts of sulfur. This particular enzyme has been linked to sulfur metabolism and H_2_S-mediated signaling pathways in earlier works. Nevertheless, since the concentration of H_2_S was not estimated in the current research, the obtained transcription pattern may be considered associated with but not caused by the H_2_S effect (Liu et al., 2024; Wang et al., 2025; Noctor et al., 2024), and the present findings provide indirect but compelling support for its involvement under Sulfur deficiency. The concomitant increase in antioxidant activity and decreased MDA content may imply a causative relationship; however, in the absence of direct measurement of endogenous H_2_S, this finding can only be regarded as associative. The observed 4.9 and 7.7-fold upregulation of the genes involved in the transport and assimilation of Sulfur is due to the strong response to the treatment with TP-5.

It is well understood that transcript abundance does not always correlate with protein abundance or enzymatic activity due to post-transcriptional regulation, translation efficiency, and protein stability (Takahashi et al., 2023; Ponomarenko et al., 2023). Therefore, the observed expression changes should be considered as correlations between gene transcription and physiology/biochemistry effects in this experiment, not as evidence that pathway is activated or enzymes are regulated (Kumar et al., 2025). Further validation through enzyme activity assays and Sulfur metabolic flux analyses will be required to confirm pathway-level regulation.

Notably, the effects of TP-5 were less apparent in 100% S conditions. This could be attributed to reduced plant dependence on microbial-assisted nutrient uptake, which has been observed in other plant-microbe interactions, where the beneficial effects are most apparent under stress conditions (Anas et al., 2025). Under conditions of sufficient Sulfur availability, the physiological requirement for rhizospheric augmentation would be reduced, possibly resulting in reduced intensity of plant-microbe interaction. Conversely, a moderate level of S limitation would result in increased plant sensitivity to microbial augmentation, thereby maximizing the benefits of symbiosis.

Despite the positive roles of the bacterial isolates in promoting plant growth during sulfur deprivation, their activities were dependent on the parameters assessed. The bacteria greatly enhanced several growths, physiological, and biochemical parameters; however, these improvements were generally less extensive and less significant compared to those elicited by the fungus *Trichoderma reseei* (TP-5). In addition, TP-5 had a broad range of impacts that covered aspects such as growth, sulfur mobilization, production of antioxidants, and gene expression involved in sulfur metabolism. This study indicates that although both sulfur-mobilizing microorganisms and fungi can play a critical role in improving soybean development during sulfur deficiency, TP-5 may be a better alternative.

## 5 Conclusion and future prospects

This research has clearly demonstrated that microbial inoculation, especially *Trichoderma reesei* (TP-5), can bring about a positive effect on soybean plant growth under 25% and 50% sulfur supply treatments. TP-5 significantly improved growth, photosynthetic pigment stability, nutrient acquisition, and yield attributes in soybean plants at 25% Sulfur supply. These physiological improvements were closely associated with enhanced redox homeostasis, as reflected by reduced oxidative damage, elevated antioxidant enzyme activities, and increased accumulation of Sulfur-containing metabolites, including cysteine, methionine, and glutathione. At the molecular level, TP-5 modulated key components of the Sulfur assimilation pathway by promoting sulfate uptake and activation while maintaining downstream metabolic balance. The coordinated regulation of Sulfur transporters, assimilatory enzymes, and hydrogen sulfide–associated signaling genes provides mechanistic support for the observed physiological and biochemical responses. Collectively, these findings indicate that TP-5 does not merely compensate for nutrient limitation but reprograms Sulfur metabolism toward greater efficiency and resilience.

Importantly, the Sulfur stress-dependent nature of TP-5 efficacy highlights the contextual specificity of plant–microbe interactions and underscores the potential of targeted bioinoculant strategies in sustainable agriculture. By improving Sulfur acquisition and utilization under moderate deficiency, TP-5 represents a promising microbial input for optimizing crop performance while reducing reliance on chemical Sulfur fertilizers. Future studies integrating enzymatic activity assays, metabolic flux analysis, and field-scale validation will further strengthen the application potential of TP-5 in Sulfur-deficient agroecosystems.

## Data Availability

The original contributions presented in the study are included in the article/**Supplementary Material**, further inquiries can be directed to the corresponding author/s.
