# Supplementary material for: Microbes-mineral interactions enhance nutrient acquisition and physiological adaptation in Glycine max under Sulfur-deficient conditions
Source: Front Plant Sci. 2026 Jul 10;17:1723856. doi: 10.3389/fpls.2026.1723856 (PMC13395674; doi:10.3389/fpls.2026.1723856)
Supplement: Supplementary file 1 [file SupplementaryFile1.docx]

Microbes-minerals interaction enhance nutrient acquisition and physiological adaptation in *Glycine max* under Sulfur deficient condition

Pratibha Verma^1,2,4,6#^, Priyanka Chauhan^1,2,6#^, Navinit Kumar^1,2^, Nishtha Mishra^1,2,3^, Sahil Mahfooz^1,7^, Vartika Gupta^5^, Aradhana Mishra^1,2*^, Lal Bahadur^2,4*^

^1^Division of Microbial Technology, CSIR-National Botanical Research Institute, Lucknow-226001, Uttar Pradesh, India.

^2^Academy of Scientific and Innovative Research (AcSIR), Ghaziabad 201002, India.

^3^Department of Chemistry, Deen Dayal Upadhyaya Gorakhpur University, Gorakhpur, India

^4^Soil Science Laboratory, CSIR–National Botanical Research Institute, Lucknow- 226001, India.

^5^Division of Environmental Technologies, CSIR–National Botanical Research Institute, Lucknow- 226001, India.

^6^School of Sciences, P P Savani University, Kosamba, Surat-394125, India

^7^Department of Industrial Microbiology, Deen Dayal Upadhyaya Gorakhpur University, Gorakhpur-273009, India

**^#^Authors contribute equally**

***Corresponding author:**

Dr. Aradhana Mishra, Senior Principal Scientist

Division of Microbial Technology, Email: [mishra.a@nbri.res.in](about:blank), [mishramyco@yahoo.com](about:blank)

CSIR- National Botanical Research Institute, Rana Pratap Marg, Lucknow, 226001, India.

Tel: 522 2297987, Fax: +91 522 220583

Dr. La Bahadur, Principal Scientist

Soil Science Laboratory, Email: lb.yadav@nbri.res.in

CSIR- National Botanical Research Institute, Rana Pratap Marg, Lucknow, 226001, India.

Tel: 0522-2297915

Legends:

**Fig S1 P-Solubilization activity of Microbial stains**


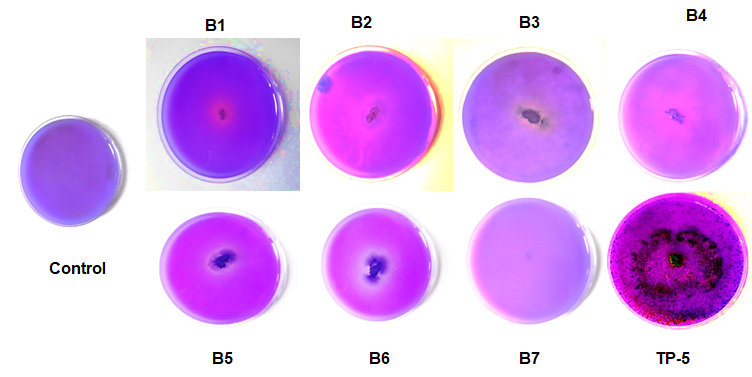


**Fig S2** Neighbor-joining phylogenetic tree analysis of 16S-rRNA of strain *Bacillus* spp. (B3) and *Brevibacillus brevis* (B5). The phylogenetic tree was generated by the maximum-likelihood method with default parameters. The scale bar corresponds to 0.5 substitutions per nucleotide position. A bootstrap analysis with 1500 replications was performed to assess group support. The GenBank accession number of each species is given after the strain’s name.


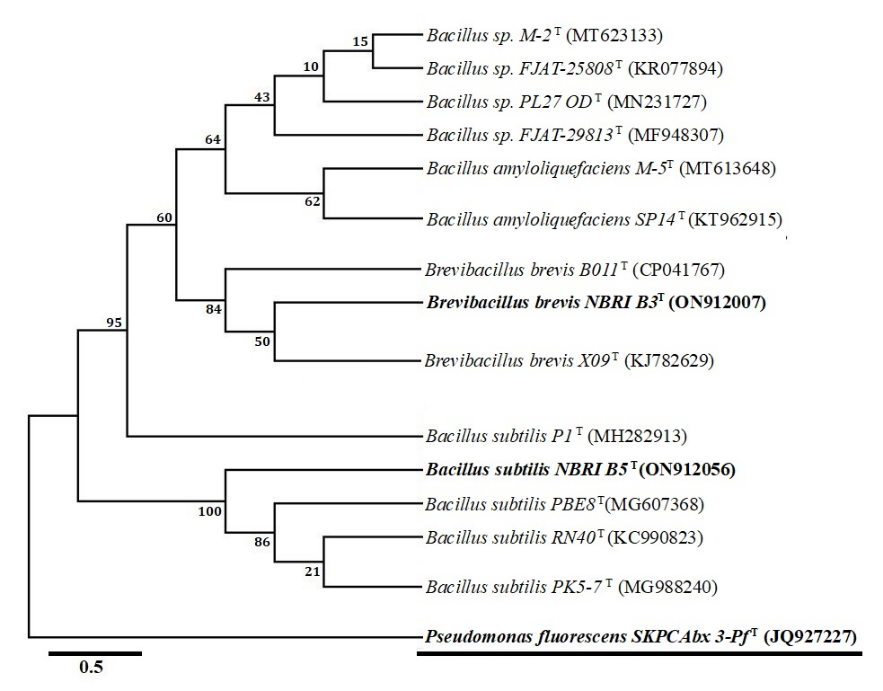


Fig S3 Greenhouse experiment of soybean plant under different S concentrations


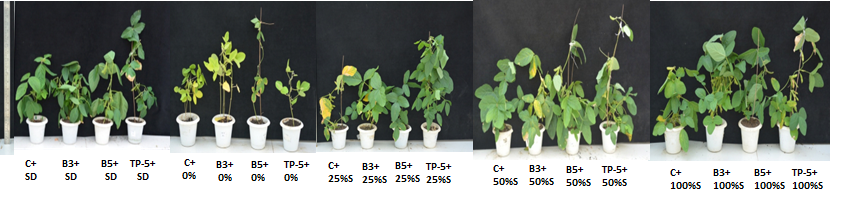


**Fig S4** Photosynthetic pigments (A) Chl ‘a’, Chl ‘b’, and total chlorophyll, (B) carotenoids, of soybean plants grown in different S concentrations in the absence and presence of different microbes (B3, B5, and TP-5) treatments. Bars represent standard errors of three replicates. The different letters indicate significant differences (P<0.05) according to Tukey’s multiple range tests.


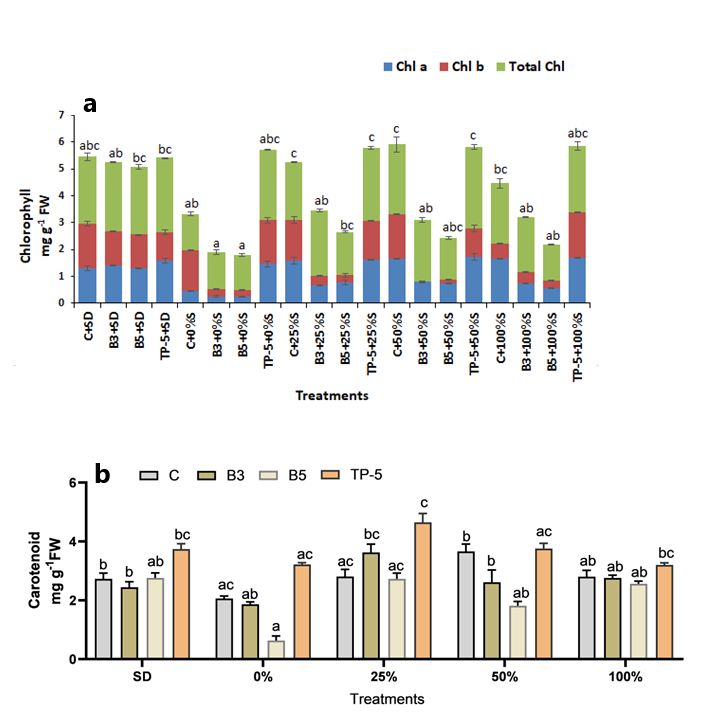


**Fig S5** Effect of non-enzymatic parameters (A,B) Total soluble sugar, and (C,D) LPX on soybean root and shoot grown in different S concentrations in the absence and presence of different microbes (B3, B5, and TP-5) treatments. Bars represent standard errors of three replicates. The different letters indicate significant differences (P<0.05) according to Tukey’s multiple range tests.


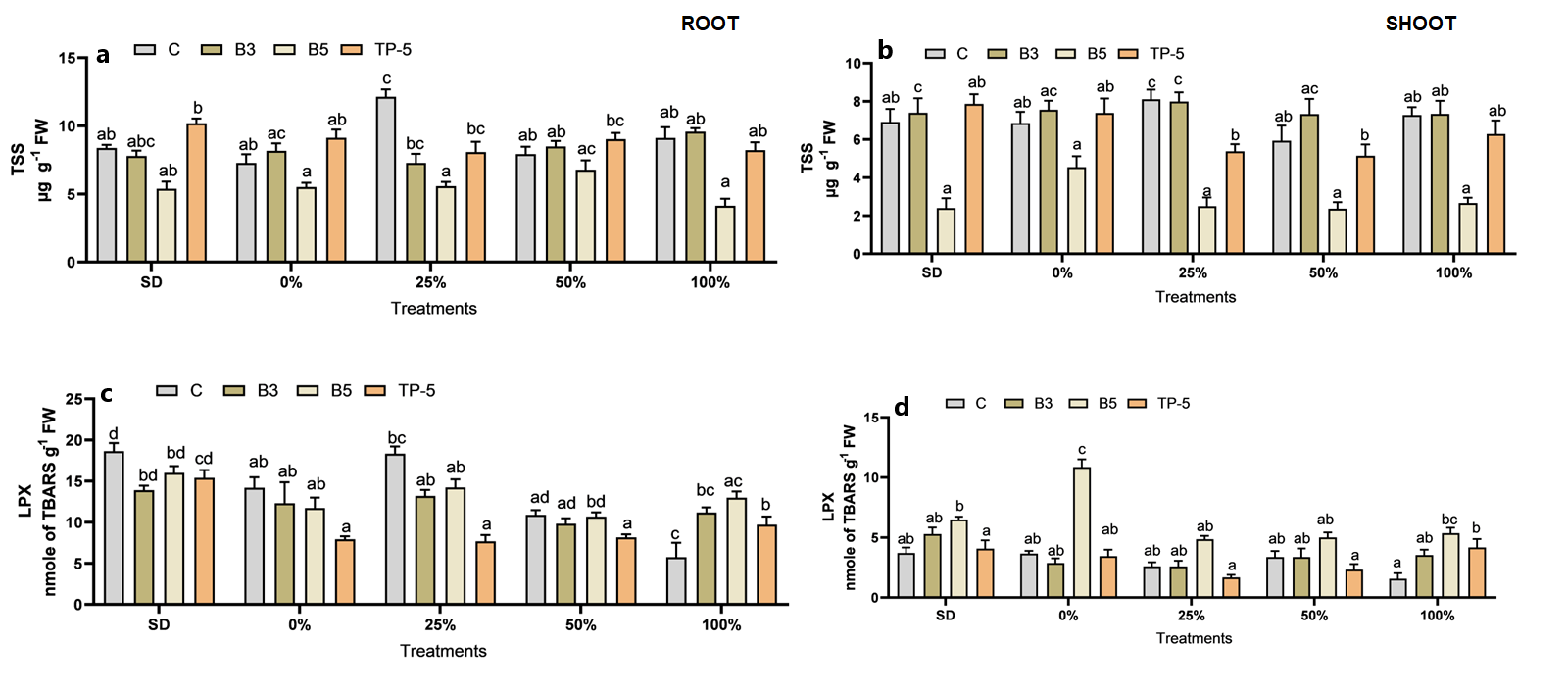


**Table S1.** Physical attributes of soybean plants grown in different Sulfur concentrations in the absence and presence of different microbes (B3, B5, and TP-5) treatments. Values are means of three replicates ± standard error (SD) indicated

| **S. No.** | **Treatments** | **Seed germination (%)** | **Root Length (cm)** | **Shoot Length (cm)** | **Fresh Weight (g)** | **Dry Weight (g)** | **Number of Pods** | **Pod Weight(g)** |
| --- | --- | --- | --- | --- | --- | --- | --- | --- |
| 1. | C+ SD | 33.33±15.77^b^ | 4.33±0.04^ab^ | 14±0.62^b^ | 6.67±1.03^b^ | 3.78±0.89^c^ | 14±1.0^b^ | 7.15±0.59^b^ |
| 2. | B3+ SD | 60±4.96^d^ | 5.33±1.24^c^ | 17.33±0.52^b^ | 5.63±0.51^ab^ | 2.55±1.03^b^ | 16.66±1.5^b^ | 11.38±1.38^bc^ |
| 3. | B5+ SD | 40±5.96^bc^ | 5.66±0.44^c^ | 14.66±1.03^b^ | 3.55±0.23^a^ | 1.74±1.86^a^ | 13±1.36^b^ | 9.56±0.38^b^ |
| 4. | TP-5+ SD | 66.66±11.90^d^ | 8.66±0.55^e^ | 22.33±0.05^c^ | 8.88±0.3^bc^ | 2.57±0.3^b^ | 22.66±1.57^bc^ | 15.57±1.38^bc^ |
| 5. | C+0% | 33.33±5.06^b^ | 5.33±0.44^c^ | 9.83±1.29^a^ | 5.03±0.44^ab^ | 1.59±1.03^a^ | 4±0.88^a^ | 1.44±1.05^a^ |
| 6. | B3+0% | 40±5.90^bc^ | 4.66±0.14^ab^ | 10.66±0.56^ab^ | 4.55±0.44 ^ab^ | 1.41±3.38^a^ | 5±0.5^a^ | 2.65±3.43^ab^ |
| 7. | B5+0% | 26.66±5.0^a^ | 4.33±0.30^ab^ | 14.66±0.61^b^ | 4.20±0.44 ^ab^ | 1.54±1.86^a^ | 3.33±0.78^a^ | 1.02±0.77^a^ |
| 8. | TP-5+0% | 66.66±5.96^d^ | 8.33±0.37^e^ | 12.33±0.46^ab^ | 7.88±0.25^b^ | 3.10±2.06^c^ | 9.66±0.2^ab^ | 6.81±1.14^b^ |
| 9. | C+25%S | 26.66±11.92^a^ | 6.33±0.45^de^ | 9.5±0.45^a^ | 4.57±0.51^ab^ | 1.03±4.03^a^ | 22.66±1.57^bc^ | 14.45±0.78^bc^ |
| 10. | B3+25% S | 46.66±14.77^c^ | 4.7±0.55^ab^ | 14.33±0.68^b^ | 15.84±0.51^c^ | 3.95±2.36^c^ | 22.66±3.6^bc^ | 15.71±1.03^bc^ |
| 11. | B5+25% S | 33.33±5.96^b^ | 6.33±0.77^de^ | 20.66±0.48^c^ | 9.09±0.51^bc^ | 3.99±0.93^c^ | 22.33±1.29^bc^ | 11.89±0.88^bc^ |
| 12. | TP-5+25% S | 80±10.02d^e^ | 12±0.51^f^ | 35.66±0.45^d^ | 25.16±0.25^e^ | 8.62±1.86^e^ | 54.33±2.54^d^ | 42.88±1.32^e^ |
| 13. | C+50% S | 33.33±5.20^b^ | 5.33±0.41^c^ | 15.33±0.34^b^ | 8.70±0.89^bc^ | 2.02±0.89^b^ | 21.33±1.65^bc^ | 15.78±0.14^bc^ |
| 14. | B3+50% S | 46.66±17.88^c^ | 4.16±0.44^ab^ | 17.33±0.48^b^ | 9.59±1.36^bc^ | 3.75±2.97^c^ | 22.66±4.01^bc^ | 16.31±0.51^c^ |
| 15. | B5+50% S | 46.66±10.32^c^ | 5.66±0.53^c^ | 20.33±0.39^c^ | 6.67±1.03^b^ | 2.88±1.54^b^ | 24.66±0.78^bc^ | 18.21±0.74^cd^ |
| 16. | TP-5+50% S | 93.33±5.0^f^ | 9.66±0.55^e^ | 14.33±0.71^b^ | 7.08±0.89^b^ | 4.84±6.01^cd^ | 34.33±2.08^cd^ | 19.8±1.50^cd^ |
| 17. | C+100% S | 46.66±4.96^c^ | 6.33±0.52^de^ | 15±0.48^b^ | 6.96±0.5^b^ | 2.17±0.51^b^ | 20.66±0.7^bc^ | 6.56±0.042^b^ |
| 18. | B3+100% S | 66.66±10.30^d^ | 3.5±0.07^a^ | 17±0.52^b^ | 10.08±0.51^bc^ | 5.86±0.68^d^ | 42.33±1.29^d^ | 28.94±0.28^d^ |
| 19. | B5+100% S | 46.66±5.16^c^ | 4.66±0.13^ab^ | 16.33±0.56^b^ | 8.61±0.51^bc^ | 4.98±1.36 ^cd^ | 48.33±2.08^d^ | 21.76±0.26^cd^ |
| 20. | TP-5+100% S | 86.66±11.02^e^ | 10.83±0.41^e^ | 33.33±0.40^d^ | 20.65±0.51^d^ | 9.63±2.25^e^ | 52.33±1.81^cd^ | 39.11±0.72^de^ |

Table S2. Details of primer pairs for nutrient related genes in *Glycine max* used for qRT-PCR data

| **Primer** | **Type** | **Primer Sequence 5'-3'** | **Gene ID** |
| --- | --- | --- | --- |
| ***ACT*** | F | TCGTATGAGCAAGGAAATTGG | AW350943 |
|  | R | TAGAGCCACCAATCCAGACAC |  |
| **ATP Sulfurylase** (***ATPS*)** | F | CTACACCTGGCTTCTTCT | LOC100805275 |
|  | R | ACTCTTGGCCTCACTTT |  |
| **Serine acetyltransferase 2 (*SAT*)** | F | TTCTCTCCCTTCCCAAAG | LOC100779412 |
|  | R | GCTAGCAACCACAACATC |  |
| **Glutathione Reductase *(GR)*** | F | CGTGAGTTTCCGATTTCTT | LOC547793 |
|  | R | TCTAGCAGTTTAGCTTGTTG |  |
| **D-cysteine desulfhydrase *(DES)*** | F | CGCCTCCTGATATGTGT | [LOC100787528](https://www.ncbi.nlm.nih.gov/gene/100787528) |
|  | R | TCCTCAAAGCCTGGTAAT |  |
| **Sulfite reductase** ***(SIR)*** | F | CGCAGCAAAGTCGAAATA | LOC547693 |
|  | R | CACGCTCCTCTCTATTGT |  |
| **Sulfate transporter 2 (*SULTR 2*)** | F | CTGTCGGGATGGGTAAT | [LOC100815818](https://www.ncbi.nlm.nih.gov/gene/100815818) |
|  | R | CTTCCACCAAACGCATAG |  |
| **Sulfate transporter 1 (*SULTR 1*)** | F | GGAAGGAGAGGTGAGTTT | [LOC100777637](https://www.ncbi.nlm.nih.gov/gene/100777637) |
|  | R | CAACGGAGAAGAATTGGAC |  |
| **Nitrite reductase (*NIR*)** | F | TTCTTGCTCCTCTTCCTC | LOC732631 |
|  | R | ATCTCTCTCCTCCACTCT |  |
